# Unspliced X-box-binding Protein 1 (XBP1) Protects Endothelial Cells from Oxidative Stress through Interaction with Histone Deacetylase 3*

**DOI:** 10.1074/jbc.M114.571984

**Published:** 2014-09-04

**Authors:** Daniel Martin, Yi Li, Junyao Yang, Gang Wang, Andriana Margariti, Zhixin Jiang, Hui Yu, Anna Zampetaki, Yanhua Hu, Qingbo Xu, Lingfang Zeng

**Affiliations:** From the ‡ Cardiovascular Division, King's College London, London SE5 9NU, United Kingdom,; the § Department of Emergency Medicine, the Second Affiliated Hospital, Xi'an Jiaotong University School of Medicine, Xi'an 710004, China,; the ¶ Centre for Experimental Medicine, School of Medicine, Dentistry and Biomedical Sciences, Queen's University Belfast, Institute of Clinical Sciences, Belfast BT12 6BL, United Kingdom,; the ‖ Centre Laboratory, 305th Hospital of the People's Liberation Army, Beijing 100017, China, and; the ** Sino-German Laboratory for Molecular Medicine, Key Laboratory for Clinical Cardiovascular Genetics, Ministry of Education, FuWai Hospital, Chinese Academy of Medical Sciences, Beijing 100037, China

**Keywords:** Cell Signaling, Endothelial Cell, Histone Deacetylase (HDAC), Oxidative Stress, Shear Stress

## Abstract

It is well known that atherosclerosis occurs geographically at branch points where disturbed flow predisposes to the development of plaque via triggering of oxidative stress and inflammatory reactions. In this study, we found that disturbed flow activated anti-oxidative reactions via up-regulating heme oxygenase 1 (HO-1) in an X-box-binding protein 1 (XBP1) and histone deacetylase 3 (HDAC3)-dependent manner. Disturbed flow concomitantly up-regulated the unspliced XBP1 (XBP1u) and HDAC3 in a VEGF receptor and PI3K/Akt-dependent manner. The presence of XBP1 was essential for the up-regulation of HDAC3 protein. Overexpression of XBP1u and/or HDAC3 activated Akt1 phosphorylation, Nrf2 protein stabilization and nuclear translocation, and HO-1 expression. Knockdown of XBP1u decreased the basal level and disturbed flow-induced Akt1 phosphorylation, Nrf2 stabilization, and HO-1 expression. Knockdown of HDAC3 ablated XBP1u-mediated effects. The mammalian target of rapamycin complex 2 (mTORC2) inhibitor, AZD2014, ablated XBP1u or HDAC3 or disturbed flow-mediated Akt1 phosphorylation, Nrf2 nuclear translocation, and HO-1 expression. Neither actinomycin D nor cycloheximide affected disturbed flow-induced up-regulation of Nrf2 protein. Knockdown of Nrf2 abolished XBP1u or HDAC3 or disturbed flow-induced HO-1 up-regulation. Co-immunoprecipitation assays demonstrated that XBP1u physically bound to HDAC3 and Akt1. The region of amino acids 201 to 323 of the HDAC3 protein was responsible for the binding to XBP1u. Double immunofluorescence staining revealed that the interactions between Akt1 and mTORC2, Akt1 and HDAC3, Akt1 and XBP1u, HDAC3, and XBP1u occurred in the cytosol. Thus, we demonstrate that XBP1u and HDAC3 exert a protective effect on disturbed flow-induced oxidative stress via up-regulation of mTORC2-dependent Akt1 phosphorylation and Nrf2-mediated HO-1 expression.

## Teaser

**Background:** The role of the unspliced XBP1 remains unclear.

**Results:** Disturbed flow concomitantly up-regulates XBP1u and HDAC3, which form a complex with Akt1 and mTOR, leading to Nrf2-mediated HO-1 expression.

**Conclusion:** XBP1u and HDAC3 synergistically exert a protective effect on disturbed flow-induced oxidative stress via regulation of HO-1 expression.

**Significance:** This study provides new insights into the physiological roles of XBP1u and HDAC3.

## Introduction

It is widely accepted that atherosclerosis occurs geographically at branch points where disturbed flow can trigger endothelial cell (EC)^4^ dysfunction via oxidative stress and inflammation (1). Under physiological conditions, cells can naturally establish homeostasis in response to different stimuli. Theoretically, the activation of oxidative stress under disturbed flow should be accompanied by the up-regulation of anti-oxidative mechanisms to maintain homeostasis within ECs. This may be a mechanism through which vascular integrity is maintained. The development of atherosclerosis could be a consequence of the disruption of the homeostasis by the intervention of other risk factors such as hyperlipidemia, diabetes, hypertension, smoking, and etc. (2, 3).

Heme oxygenase 1 (HO-1) is an inducible isoform of the heme oxygenase family, which catalyzes the degradation of heme, producing biliverdin, iron, and carbon monoxide (4). HO-1 was originally identified as a 32-kDa stress responsive protein to UV-irradiation, hydrogen peroxide, and sodium arsenite (5). HO-1 is induced ubiquitously in cells in response to oxidative stress, hypoxia, heavy metal ions, cytokines, glutathione depletion, and etc. (6). The induction of HO-1 and the derived carbon monoxide plays a protective role against cell apoptosis (7). Disruption of the HO-1gene (*HMOX-1*) does not affect mouse survival but increases end-organ damage and mortality during endotoxemia due to increased oxidative stress (8). In EC, HO-1 can be induced by both laminar flow and disturbed flow through oxidative stress and Nrf2 (NF-E2-related factor 2) activation (9). However, the existence of a signaling pathway between the mechanosensor and Nrf2-mediated HO-1 expression remains unknown.

The X-box binding protein 1 (XBP1) is also a stress responsive gene. In contrast to most stress responsive genes, XBP1 mRNA undergoes alternative splicing via inositol-requiring enzyme 1 α (IRE1α). This occurs in response to endoplasmic reticulum (ER) stress, resulting in an open reading frameshift (10, 11). XBP1 protein exists as 29-kDa unspliced (XBP1u) and 56-kDa spliced (XBP1s) isoforms. Both isoforms have an identical N-terminal dimerization domain and internal DNA binding domain but differ in the C terminus. XBP1s contains a transcriptional activation domain in the C terminus and functions as an intact transcription factor (10). The majority of the previously described XBP1 functions are assigned to XBP1s. Our previous studies have demonstrated that XBP1s plays multiple roles in EC proliferation, autophagy response, and apoptosis (12–14). The C terminus of XBP1u contains a signal for proteasome-mediated degradation, negatively regulating XBP1s function (15). There remains very little investigation into the role of XBP1u compared with XBP1s.

Histone deacetylase 3 (HDAC3) is a class I HDAC (16). Disruption of the *HDAC3* gene is lethal at an early embryonic stage (17). It is reported that cigarette smoke reduces HDAC3 activity via posttranslational modification (18), which is the first indirect evidence that HDAC3 is involved in response to oxidative stress. Our previous study provides direct evidence that up-regulation of HDAC3 by disturbed flow is essential for EC survival under oxidative stress via activation of Akt phosphorylation (19). HDAC3 deficiency in ECs accelerates vessel injury-induced neointima formation. Our studies have also demonstrated that HDAC3 homeostasis is essential for EC differentiation from stem/progenitor cells (20, 21), inflammatory reactions (22), and endothelial-to-mesenchymal transition (23). In this study, we found that HDAC3 cooperated with XBP1u to modulate HO-1 expression in response to disturbed flow. To scrutinize the molecular mechanisms of this process, the present study aims to clarify the role of XBP1 interaction with the partners in maintaining endothelial functions. We demonstrated that an interaction between XBP1 and HADC3 resulted in PI3K/Akt1 activation and HO-1 expression. This process is crucial for endothelial survival in response to oxidative stress.

## EXPERIMENTAL PROCEDURES

### 

#### 

##### Materials

All cell culture medium and serum were purchased from Invitrogen, whereas cell culture supplements were purchased from Sigma. Antibodies against XBP1 (sc-7160), HDAC3 (sc-136290) phospho-Akt (sc-7985R), Akt1 (sc-1619), Nrf2 (sc-722), mTOR (sc-1549), histone H3 (sc-10809), IRE1α (sc-20790), and GAPDH (sc-25778) were purchased from Santa Cruz Biotechnology; antibodies against FLAG (F2426, F1804, and F7425), HA (H6908) and tubulin (T8203) were from Sigma; antibody against HO-1 (ab13248) was purchased from Abcam. Antibodies against XBP1u and XBP1s were raised in rabbits with peptides CRSSQRSTQKDPVPY and DSGGIDSSDSESDIC, respectively, by GenScript Corp. All secondary antibodies were from DakoCytomation. Inhibitors LY294002, PD98059, SU5416, actinomycin D, cycloheximide, and Tin protoporphyrin IX were purchased from Sigma. AZD2014 was purchased from Selleckchem. All inhibitors were dissolved in DMSO. All other chemicals were also from Sigma.

##### Cell Culture

Human umbilical vein ECs (HUVECs) were cultured on 0.04% gelatin-coated flasks in M199 medium supplemented with 1 ng/ml β-EC growth factor, 3 μg/ml EC growth supplement from bovine neural tissue, 10μ/ml heparin, 1.25 μg/ml thymidine, 10% fetal bovine serum (FBS), 100 μ/ml penicillin, and streptomycin in a humidified incubator supplemented with 5% CO_2_. The cells were split every 3 days at a ratio of 1:3. Cells up to passage 10 were used in this study. HEK293 cells were maintained in DMEM supplemented with 10% FBS and penicillin/streptomycin and were split every 3 days at a ratio of 1:4. Mouse embryonic fibroblasts were isolated from *XBP1*^+/−^ cross-bred embryonic day 8.5 embryos (12), cultured in DMEM supplemented with 10% FBS and penicillin/streptomycin. The genotype of the cells was verified by PCR with primer triplet of P1 (5′-atcctgtcttgaaatggcaagtgttgg-3′), P2 (5′-tggcaaggctgagcctgatcg-3′), and P3 (5′-ggaactagagataccactgag-3′), giving rise to a 265-bp band for wild type and a 365-bp band for *XBP1*^−/−^ homozygous and double bands for *XBP1*^+/−^ heterozygous.

##### Disturbed Flow

Flow experiments were performed exactly as described previously (14). Briefly, the disturbed flow was created by placing the flask on a platform shaker (Labnet, model Rocker 25) with parameters of 2.0-mm culture medium depth, 10-cm length flask, ± 7° rotating angle and frequency of 0.5 Hz (*i.e.* 2 s per cycle), respectively. Unshaken cells were kept for same duration as static control. For inhibitor assays, the inhibitors were included in culture medium for 1 h prior to flow and maintained during the flow process.

##### 3-(4,5-Dimethylthiazol-2-yl)-2,5-diphenyltetrazolium Bromide Cell Proliferation Assay

HUVECs were challenged with 50 μmol/liter H_2_O_2_ for 24 h after 24 h post-infection with Ad-null or Ad-*XBP1u* virus at 10 MOI or with 20 μmol/liter H_2_O_2_ for 24 h after 72 h post-infection with non-target shRNA or *XBP1* shRNA lentivirus at 10 MOI, followed by 3-(4,5-dimethylthiazol-2-yl)-2,5-diphenyltetrazolium bromide cell proliferation assay with the CellTiter 96 Aqueous One Solution Cell Proliferation assay kit according to the protocol provided (Promega). Briefly, 3-(4,5-dimethylthiazol-2-yl)-2,5-diphenyltetrazolium bromide reagent was diluted at 1:4 with M199 containing 2% FBS, applied to HUVECs at 300 μl/well in 24-well plate and incubated at 37 °C for 30 min to 2 h. 300 μl/well of 0.2% SDS was then added to stop the reaction. The absorbance at *A*_490 nm_ was measured with a laminator luminometer. The relative cell survival was defined as the ratio of *A*_490 nm_ of the test group to that of control group with that of control group set as 1.0.

##### Cell Apoptosis Analysis

Mouse embryonic fibroblasts were challenged with 50 μmol/liter H_2_O_2_ for 24 h, followed by apoptosis analysis using the Apo-Direct flow cytometry kit (Chemicon) with protocol provided. PBS was included as control. Briefly, the treated cells were detached with trypsin and fixed in solution with 1% paraformaldehyde on ice for 1 h and washed three times with PBS. The cell pellet was resuspended in 50 μl of staining solution (TdT reaction buffer, TdT enzyme, fluorescein-dUTP) and incubated for 60 min at 37 °C, with shaking every 15 min. The reaction was stopped through the addition of 1 ml of rinse buffer and washed three times with the rinse buffer. The cell pellet was resuspended in 500 μl of propidium iodide/RNase A solution and incubated in the dark at room temperature for 30 min, followed by flow cytometry analysis of fluorescence at 520 nm with a 488-nm Argon laser.

##### Ex Vivo Experiments

The artery *ex vivo* survival experiments were performed as described previously (14). Briefly, arteries were isolated from *Tie2-LacZ/ApoE*^−/−^ mice and cut into an ∼2-mm^2^ segment. The segments were incubated with 0.5 ml of M199 medium plus 15% FBS containing no virus (control), empty virus (Ad-null), or Ad-*XBP1u* virus at 1 × 10^6^ plaque-forming unit/ml in 24-well plate for 6 h. Three segments were included in each group. The virus solutions were then removed, and 1 ml of fresh medium was added to each well. Twenty-four hour post infection, the segments were treated with PBS or 50 μmol/liter H_2_O_2_ for 24 h. The segments were fixed with 4% formaldehyde and 1% glutaraldehyde in PBS for 5 min, followed by X-gal staining overnight (24). The segments were mounted on slide with vessel lumen face up. Images were assessed by Zeiss Axioplan 2 Imaging microscope with Plan-NEOPLUAR 20×/0.5 objective lenses, AxioCam camera and Axiovision software at room temperature and were processed by Adobe Photoshop software. Cell numbers were counted under the microscope. The relative cell number was defined as the ratio of cell numbers/mm^2^ of virus-infected group to that of uninfected control group with that of control set as 1.0.

##### Plasmid Cloning and Luciferase Activity Assay

A 700-bp mouse *HDAC3* promoter fragment was amplified by PCR using a primer set of 5′-gacactctcgagaatgcctactcgcgttgc-3′ and 5′-gtgaccaagcttcgagcctcagctgcc-3′, cloned into the XhoI/HindIII sites of pGL3-Luc (Promega), and verified by DNA sequencing. The resulting plasmid was designated as *HDAC3*-Luc. The *XBP1u* cDNA sequence was amplified by RT-PCR with a primer set of 5′-ggagctggtaccctggtggtggtggcagcc-3′ and 5′-tctgagaagcttacagtattggatcattcc-3′, cloned into the KpnI/HindIII site of pCMV5-HA vector, and verified by DNA sequencing. The *XBP1u* open reading frame is fused to HA tag at the N terminus, designated as pCMV5-HA-*XBP1u*. The cloning of other plasmids, pShuttle2-FLAG-*XBP1s*, pShuttle2-FLAG-*XBP1u*, pShuttle2-FLAG-*HDAC3*, and the *HDAC3* mutant variants has been described in previous reports (14, 19).

HUVECs were seeded in 12-well plates at 5 × 10^4^ cells/well 24 h prior to transfection. *HDAC3*-Luc (0.1 μg/well) vector was co-transfected with 0.1 μg/well pShuttle2-FLAG-*XBP1s* or -*XBP1u* expression vector into HUVECs with FuGENE 6 (Roche Applied Science). pGL3-Luc basic vector and grp78-luc vector were included as negative and positive luciferase vector control, respectively. pShuttle2-lacZ vector was used as an empty vector control. *Renilla*-Luc (0.05 μg/well) was included as an internal control. Forty-eight hours later, firefly and *Renilla* luciferase activity was assessed with respective assay kit (Promega). The relative luciferase activity was defined as the ratio of readout for firefly luciferase to that for *Renilla* luciferase with that of control group set as 1.0.

##### Quantitative PCR

Total cellular RNA was extracted using Qiagen RNeasy kit according to the protocol provided. Two microgram RNA was transcribed into cDNA using Improm-II reverse transcription system (Promega). Twenty nanogram cDNA (relative to RNA amount) was amplified by PCR with cyber green solution (Applied Biosystems) in a Sequence Detection System 7000 (Applied Biosystems). The primers were as follows: *XBP1*, 5′-agcactcagactacgtgcacct-3′ and 5′-tgcccaacag gatatcagactc-3′; *HDAC3*, 5′-catctctgctggtagaagagg-3′ and 5′-catcatagaactcattgggtg-3′; *HMOX-1*, 5′-gccagcaacaaagtgcaagatt-3′ and 5′-tgagtgtaaggacccatcggag-3′; *Nrf2*, 5′-cagtggatctgccaactactc-3′ and 5′-tggagaggatgctgctgaagg-3′; and *18s*, 5′-cccagtaagtgcgggtcataa-3′ and 5′-ccgagggcctcactaaacc-3′.

##### Adenoviral and shRNA Lentiviral Infection

For adenoviral infection, HUVECs were incubated with Ad-null, Ad-*HDAC3*, or Ad-*XBP1u* virus at 10 MOI for 6 h and then cultured in fresh complete growth medium for time duration indicated in figure legends. For shRNA lentiviral infection, HUVECs were incubated with 100 transduction unit/cell of non-target shRNA or *XBP1* shRNA or *IRE1*α shRNA or *HDAC3* shRNA lentiviruses in the presence of 10 mg/ml polybrene for 16 h, followed by culture in fresh complete growth medium for 72 h, and subjected to further treatments.

##### siRNA Transfection

Human *Nrf2* siRNA (sc-37030) was purchased from Santa Cruz Biotechnology and reconstituted accordingly. For siRNA transfection assay, HUVECs in 75-ml flasks were transfected with 50 μl of 10 μmol/liter control siRNA or Nrf2 siRNA together with 50 μl of Lipofectamine RNAimax (Invitrogen) according to protocol provided. For virus infection assays, 48 h post-transfection, the cells were infected with 10 MOI Ad-null, Ad-*XBP1u* or Ad-*HDAC3* viruses and incubated for 24 h, followed by Western blot analysis. For shear stress assays, 72 h post-transfection, the cells were subjected to disturbed flow for 4 h, followed by Western blot analysis.

##### Immunoprecipitation, Immunoblotting, and Immunofluorescence Staining

Immunoprecipitation and immunoblotting were performed according to standard procedures described elsewhere. One milligram lysate was used for immunoprecipitation, whereas 25 μg was used for input or direct immunoblotting. For unconjugated antibody, 2 μg of antibody and 10 μl of protein G beads (Sigma) were used for one immunoprecipitation assay. For agarose-conjugated antibody, 10 μl of such beads were directly used for each immunoprecipitation assay. Immunofluorescence staining was performed using standard procedures. Briefly, adenovirus-infected or uninfected HUVECs were seeded on 0.04% gelatin-coated glass slides with or without flow treatment. The cells were fixed with methanol and permeabilized with 0.1% Triton X-100, blocked with 5% normal swine serum, incubated with primary antibodies, followed by incubation with Alexa Fluor 488- or 594-labeled secondary antibodies and counterstaining with DAPI. Images were taken by using SP5 confocal microscope (Leica) and were processed by Adobe Photoshop software. Magnification was indicated in figures.

##### Cellular Fractionation

HUVECs were infected with Ad-null, Ad-*XBP1u*, or Ad-*HDAC3* at 10 MOI for 6 h and incubated for another 18 h. Fresh medium containing DMSO or 5 μmol/liter AZD2014 were added and incubated for 24 h. The cytosol and nuclear extracts were harvested with procedures described previously (12).

##### Chromatin Immunoprecipitation

Chromatin immunoprecipitation was performed with ChIP assay kit (17-295, Millipore) according to the protocol provided. Briefly, HUVECs were subjected to disturbed flow for 2 h, followed by the ChIP assay. Rabbit anti-XBP1u and anti-XBP1s were used, and normal rabbit IgG was included as negative control. Six sets of primer pairs were used to cover the 1.5-kb HDAC3 promoter region (GenBank^TM^ accession no. AB457579.1). The sequences include the following: position +1 ∼ −276, 5′-ccacggtcttggccatggtgc-3′ *versus* 5′-tcggcttcccgaggatctgac-3′; position −276 ∼ −520, 5′-tcagatcctcgggaagccgag-3′ *versus* 5′-gtttgggtccgggtaggggac-3′; position −520 ∼ −720, 5′-cccctacccggacccaaactc-3′ *versus* 5′-gctgagagcggtggcaggctc-3′; position −720 ∼ −960, 5′-gagcctgccaccgctctcagc-3′ *versus* 5′-ttctcccaccctgaccacctg-3′; position −960 ∼ −1195, 5′-gccaggtggtcagggtgggag-3′ *versus* 5′-agctctctaccacgaccatgg-3′; position −1195 ∼ −1467, 5′-accatggtcgtggtagagagc-3′ *versus* 5′-aagagcatatatagcccatgttgg-3′. Routine PCR was performed using these primer sets to amplified XBP1u- or XBP1s-bound DNA fragment.

##### Statistical Analysis

Data expressed as the mean ± S.E. were analyzed using GraphPad Prism software (version 5) with *t* test for pair-wise comparisons or analysis of variance, when *t* test was inappropriate, followed by Dunnett's multiple comparison tests, and significance was depicted by asterisks (*, *p* < 0.05).

## RESULTS

### 

#### 

##### Disturbed Flow Activates XBP1u in a Similar Manner to HDAC3

**FIGURE 1.**
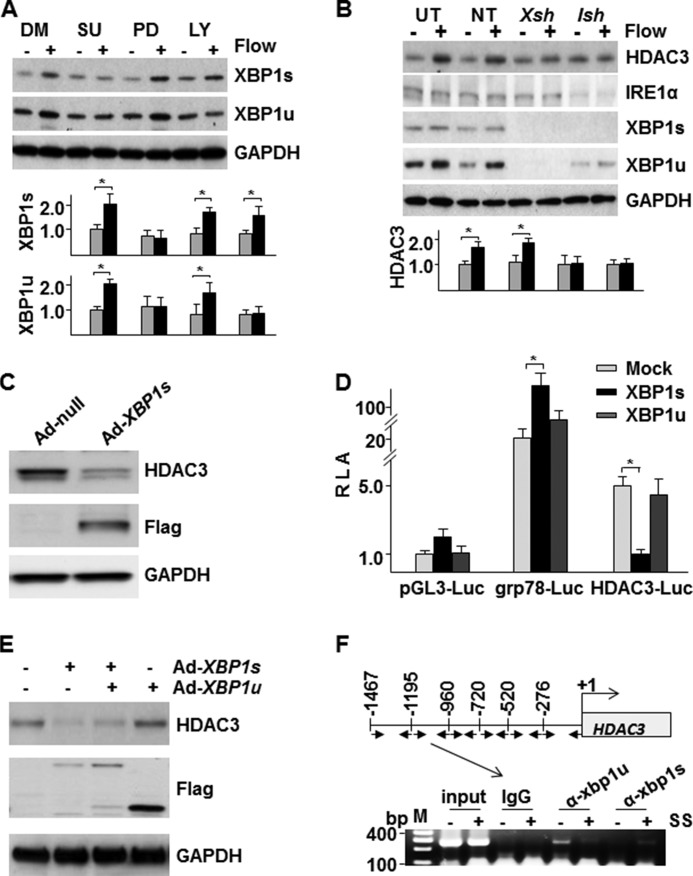
**XBP1u protein was essential for disturbed flow-induced HDAC3 up-regulation.** *A*, VEGF-PI3K/Akt pathway was involved in disturbed flow (4 h)-induced up-regulation of *XBP1* expression and splicing. *DM*, DMSO (vehicle control); *SU*, SU5416 (1 μmol/liter, VEGF receptor inhibitor); *PD*, PD98059 (5 μmol/liter, ERK inhibitor); *LY*, LY294002 (5 μmol/liter, PI3K/Akt inhibitor). *B*, knockdown of *XBP1* or *IRE1*α abolished disturbed flow (4 h)-induced HDAC3 up-regulation. *UT*, untransfected; *NT*, non-target shRNA transfected; *Xsh*, *XBP1* shRNA transfected; *Ish*, *IRE1*α shRNA transfected. Disturbed flow was applied to HUVECs 72 h post transfection for 4 h. *C*, Overexpression of spliced XBP1 down-regulated HDAC3 protein. FLAG indicates the exogenous XBP1s protein. *D*, spliced XBP1 suppressed *HDAC3* gene transcription. *RLA*, relative luciferase activity. pGL3-luc basic vector was included as negative control, whereas grp78-Luc vector was used as positive control. Mock, pShuttle-*LacZ* plasmid; XBP1s, pShuttle-FLAG-*XBP1s* plasmid; *XBP1u*, pShuttle-FLAG-*XBP1u* plasmid. *E*, XBP1u antagonized XBP1s on the regulation of HDAC3 protein. HUVECs were co-infected with Ad-*XBP1s* and Ad-*XBP1u* at 10 MOI each for 48 h. Ad-null was included as control and to compensate the MOI. FLAG indicates the exogenous XBP1s and XBP1u proteins. *F*, XBP1u and XBP1s differentially bound to HDAC3 promoter in response to disturbed flow. ChIP assay was performed to analyze the binding of XBP1u and XBP1s to the HDAC3 promoter in static and disturbed flow-treated HUVECs (4 h). Six sets of primer pairs covered the +1 ∼ −1467 region (*upper panel*), and PCR showed that XBP1u and XBP1s differentially bound to −960 ∼ −1195 region in response to disturbed flow (*lower panel*). *SS*, shear stress. Data presented are representative or average of three independent experiments. *, *p* < 0.05.

Our previous studies have demonstrated that disturbed flow sustainably activates *XBP1* expression and splicing (14) and that disturbed flow activates HDAC3 in a KDR/PI3K-Akt pathway-dependent manner (19). In this study, we found that disturbed flow-induced up-regulation of XBP1u was ablated by the presence of KDR inhibitor SU5416 and PI3K/Akt inhibitor LY294002, whereas *XBP1* splicing was only ablated by SU5416 (Fig. 1*A*). This suggests that XBP1u was regulated by a similar mechanism to HDAC3 (19). As disturbed flow concomitantly up-regulated HDAC3, XBP1u, and XBP1s, we wondered whether there was cross-talk between HDAC3 and both XBP1 isoforms. XBP1s is produced by IRE1α activation (11) and down-regulation of XBP1s can be achieved by knockdown of IRE1α. Knockdown of *XBP1* or *IRE1*α abolished disturbed flow-induced HDAC3 up-regulation (Fig. 1*B*), indicating that there is relationship between both XBP1 isoforms and HDAC3 under disturbed flow. To further examine the involvement of XBP1s in flow-induced HDAC3 up-regulation, exogenous overexpression of XBP1s was introduced into HUVECs via adenoviral gene transfer. As shown in Fig. 1*C*, overexpression of XBP1s actually decreased HDAC3 protein due to transcriptional repression as revealed by the *HDAC3*-Luc reporter analysis (Fig. 1*D*). Overexpression of XBP1u had no effect on *HDAC3* transcription (Fig. 1*D*) but antagonized the effect of XBP1s and protected HDAC3 protein levels (Fig. 1*E*). A ChIP assay revealed that both XBP1u and XBP1s could bind to the −960 ∼ −1195 region of *HDAC3* promoter (Fig. 1*F*). Under static condition, more XBP1u bound to this region, whereas during disturbed flow, more XBP1s bound to this region. These results suggest that there may be a cross-talk between HDAC3 and XBP1u.

##### XBP1u Level Is Related to EC Survival under Oxidative Stress

**FIGURE 2.**
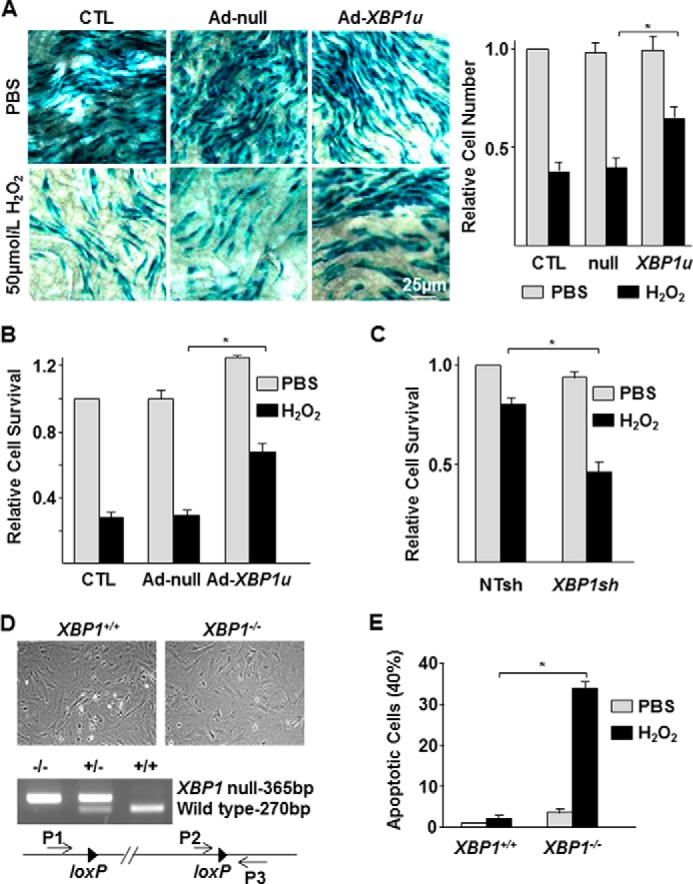
**XBP1u protected cell survival under oxidative stress.** *A*, overexpression of XBP1u increased EC survival *ex vivo* under 50 μmol/liter H_2_O_2_. The *left panel* shows the X-gal staining images, whereas the *right panel* indicates the relative cell numbers that were defined as cells/mm^2^ with that of uninfected (*CTL*)/PBS group set as 1.0. *B*, overexpression of XBP1u attenuated H_2_O_2_-induced cell loss in HUVECs. *C*, knockdown of *XBP1* enhanced H_2_O_2_ (20 μmol/liter)-induced cell loss in HUVECs. *D*, H_2_O_2_ induced significant cell apoptosis in *XBP1*^−/−^ mouse embryonic fibroblasts. The *left panel* shows the morphology of mouse embryonic fibroblasts isolated from wild type (*XBP1*^+/+^) and *XBP1*-null (*XBP1*^−/−^) embryos and the PCR strategy to verify the disruption of the *XBP1* gene. The *right panel* indicates the effect of 20 μmol/liter H_2_O_2_ on cell apoptosis. Data presented are representatives or average of three independent experiments. *, *p* < 0.05.

HDAC3 has been demonstrated to protect cells from oxidative stress (19, 25). To assess whether up-regulation of XBP1u has a similar protective effect, arterial segments were isolated from *Tie2-LacZ/ApoE*^−/−^ mice and infected with Ad-null or Ad-*XBP1u* viruses followed by 50 μmol/liter H_2_O_2_ challenge. In these mice, the β-galactosidase is selectively expressed in endothelial cells and some progenitor cells driven by the *Tie2* promoter. X-gal staining reveals the endothelium. Overexpression of XBP1u significantly reduced H_2_O_2_-induced EC loss from the vessel wall (Fig. 2*A*), which was further confirmed by *in vitro* experiments challenging HUVECs with 50 μmol/liter H_2_O_2_ (Fig. 2*B*). In contrast, knockdown of *XBP1* via shRNA lentiviral infection slightly increased the basal level of cell apoptosis but significantly augmented H_2_O_2_-induced HUVECs apoptosis even at a low concentration (20 μmol/liter) (Fig. 2*C*). Wild type and *XBP1* null (*XBP1*^−/−^) embryonic fibroblasts were isolated from *XBP1*^+/−^ cross-bred mouse embryonic day 8.5 embryos and verified by PCR (Fig. 2*D*). Spontaneously apoptotic cells were higher in *XBP1* null cells than that in wild type cells (4% *versus* 1%), which dramatically increased after 20 μmol/liter H_2_O_2_ challenge (35% *versus* 2.5%, Fig. 2*E*). These results suggest that XBP1u is essential for EC survival especially under oxidative stress.

##### XBP1u and HDAC3 Activates HO-1 in an Nrf2-dependent Manner

**FIGURE 3.**
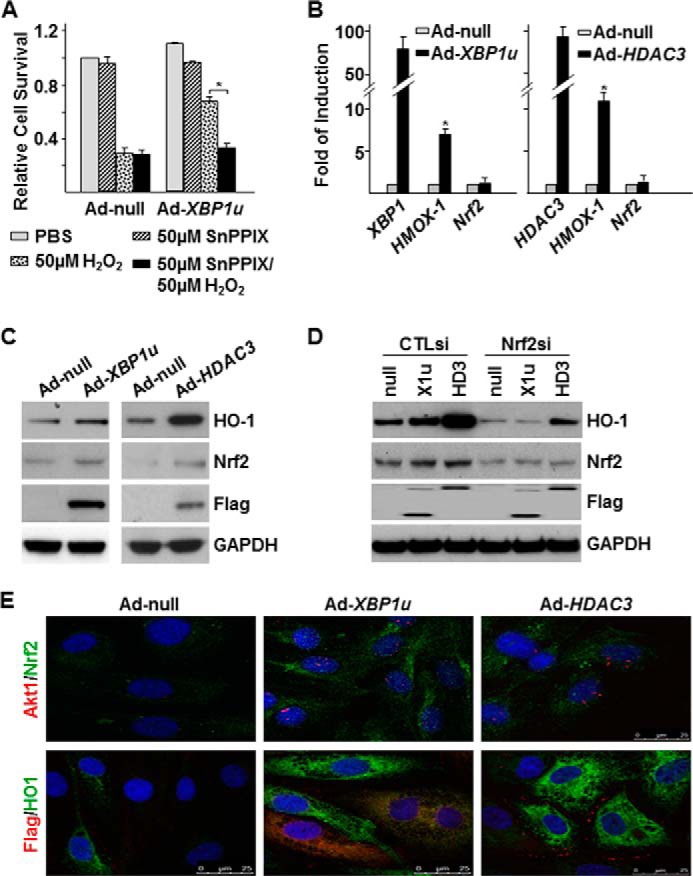
**XBP1u-mediated cell survival was through regulation of HO-1 expression.** *A*, HO-1 inhibitor SnPPIX abolished the protective effect of XBP1u overexpression on cell survival under H_2_O_2_ challenging. *B*, quantitative RT-PCR revealed that over-expression of *XBP1u* or *HDAC3* up-regulated *HMOX-1* mRNA level without effect on *Nrf2* mRNA level. *C*, Western blot analysis showed that overexpression of XBP1u or HDAC3 up-regulated both Nrf2 and HO-1 protein levels. *D*, knockdown of Nrf2 abolished XBP1u or HDAC3-induced HO-1 expression. *CTLsi*, control siRNA. *E*, overexpression of XBP1u or HDAC3 increased Nrf2 nuclear translocation and HO-1 expression in the infected and adjacent cells. Double immunofluorescence staining was performed with anti-Akt1 (*red*) and anti-Nrf2 (*green*) antibodies or with anti-FLAG (*red*) for exogenous XBP1u or HDAC3 and anti-HO-1 (*green*) antibodies. Data presented are representatives or average from three independent experiments. *, *p* < 0.05.

The degradation of heme produces biliverdin, ion, and carbon monoxide, in which HO-1 plays a rate-limiting role (4). It has been reported that HO-1 protects human ECs and vascular SMCs survival under H_2_O_2_ challenge (26, 27). Therefore, we wondered whether the increase of XBP1u-induced cell survival under H_2_O_2_ challenge was due to HO-1. To test this, the HO-1 inhibitor, Tin protoporphyrin IX (28), was included in H_2_O_2_ challenge experiments. Indeed, the presence of Tin protoporphyrin IX abolished XBP1u-mediated cell survival (Fig. 3*A*), suggesting that XBP1u promotes EC survival under oxidative stress via HO-1. Further experiments revealed that the overexpression of either XBP1u or HDAC3 up-regulated *HMOX-1* gene expression at the mRNA (Fig. 3*B*) and protein (Fig. 3*C*) levels. The mRNA level of the *HMOX-1* upstream transcription factor Nrf2 (29) remained unchanged (Fig. 3*B*), but the protein level was significantly up-regulated by XBP1u or HDAC3 (Fig. 3*C*), which might be through post-translational stabilization (30). Knockdown of *Nrf2* by siRNA abolished Ad-*XBP1u*-induced and significantly attenuated Ad-*HDAC3*-induced HO-1 proteins (Fig. 3*D*). Immunofluorescence staining revealed that overexpression of XBP1u or HDAC3 increased the nuclear localization of Nrf2 protein (Fig. 3*E*). Importantly, overexpression of XBP1u or HDAC3 not only increased HO-1 protein in the infected cells but also in adjacent cells (Fig. 3*E*), suggesting that some secreted factors are involved. These results suggest that XBP1u or HDAC3 promotes EC survival under oxidative stress through Nrf2-mediated HO-1 expression.

##### XBP1u and HDAC3 Activate Akt1 Phosphorylation via mTOR Complex

**FIGURE 4.**
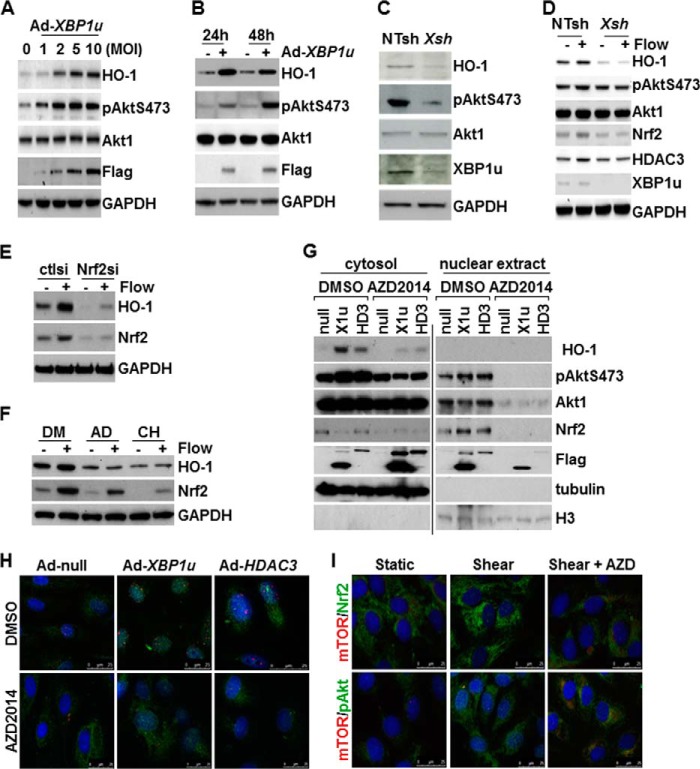
**XBP1 was essential for disturbed flow-induced up-regulation of HO-1.** *A*, overexpression of XBP1u up-regulated HO-1 and Akt phosphorylation in a dose-dependent manner. HUVECs were infected with Ad-*XBP1u* at MOI indicated for 24 h, followed by Western blot analysis. Ad-null was included to compensate the MOI. *B*, overexpression of XBP1u maintained a high level of Akt1 phosphorylation and HO-1 expression. HUVECs were infected with Ad-*XBP1u* at 10 MOI for 24 h and 48 h, followed by Western blot analysis. Ad-null was included as control. FLAG indicates the exogenous XBP1u. *C*, knockdown of *XBP1* via shRNA lentivirus (*XBP1sh*) decreased basal level of Akt1 phosphorylation and HO-1 expression. Non-target shRNA lentivirus (*NTsh*) was included as control. *D*, knockdown of *XBP1* via shRNA lentivirus (*XBP1sh*) abolished disturbed flow-induced HO-1 expression. Non-target shRNA lentivirus (*NTsh*) was included as control. *E*, Nrf2 was necessary for flow-induced HO-1 expression. HUVECs were transfected with control siRNA (*CTLsi*) or Nrf2 siRNA (*Nrf2si*) for 72 h, followed by disturbed flow for 4 h. *F*, flow stabilized Nrf2 via post-translational modification. HUVECs were treated with 1 μmol/liter actinomycin D (*AD*) or 30 mg/liter cycloheximide (*CH*) for 1 h, followed by disturbed flow for 4 h or kept at static conditions in the presence of the inhibitors. DMSO (*DM*) was included as vehicle control. *G*, AZD2014 abolished Ad-XBP1u (*X1u*) or Ad-HDAC3 (*HD3*)-induced pAkt Ser-473 phosphorylation, Nrf2 nuclear translocation, and HO-1 expression. HUVECs were infected with Ad-null or Ad-*XBP1u or Ad-HDAC3* at 10 MOI for 24 h and then treated with 5 μmol/liter AZD2014 for 24 h, followed by cellular fraction isolation and Western blot analysis. DMSO was included as vehicle control. The anti-FLAG antibody was included to detect exogenous XBP1u and HDAC3. Antibodies against α-tubulin and histone H3 were included to indicate cytosol and nuclear extract, respectively. The samples from cytosol and nuclear extraction were run on separate gels but performed Western blot at the same time and exposed to x-ray film exactly at the same time period. *H*, AZD2014 attenuated XBP1u/HDAC3-induced Akt1 phosphorylation in nucleus. HUVECs were infected with Ad-null or Ad-*XBP1u or Ad-HDAC3* at 10 MOI for 24 h and then treated with 5 μmol/liter AZD2014 for 24 h, followed by double immunofluorescence staining with anti-mTOR (*red*) and anti-pAkt Ser-473 (*green*) antibodies. *I*, AZD2014 reduced flow-induced Nrf2 nuclear translocation. HUVECs were treated with 5 μmol/liter AZD2014 for 1 h, followed by disturbed flow for 4 h or being kept at static conditions in the presence of ZAD2014. DMSO was included as vehicle control. Double immunofluorescence staining was performed with anti-mTOR (*red*) and anti-Nrf2 (*green*) or pAkt Ser-473 (*green*) antibodies. Data presented are representatives of three independent experiments.

The PI3K/Akt pathway plays a key role in HO-1 expression (31). Our previous study has demonstrated that overexpression of HDAC3 increases Akt phosphorylation (19). In this study, Western blot analysis revealed that overexpression of XBP1u induced simultaneous increase in Akt phosphorylation and HO-1 protein in a dose- and time-dependent manner (Fig. 4, *A* and *B*). Knockdown of *XBP1* decreased the basal level of Akt phosphorylation and HO-1 protein (Fig. 4*C*). Disturbed flow is reported to activate HO-1 expression (32). We also detected the up-regulation of HO-1, Nrf2, and Akt1 phosphorylation by disturbed flow (Fig. 4*D*). As expected, these effects were totally abolished by *XBP1* knockdown via shRNA lentiviral infection (Fig. 4*D*). Further experiments confirmed that Nrf2 was necessary for flow-induced HO-1 up-regulation, as siRNA-mediated knockdown of Nrf2 abolished flow-induced HO-1 expression (Fig. 4*E*). The addition of the transcription inhibitor (actinomycin D) or translation inhibitor (cycloheximide) also abolished flow-induced HO-1 up-regulation (Fig. 4*F*). The addition of actinomycin D and especially cycloheximide reduced the basal level of Nrf2. However, disturbed flow still up-regulated Nrf2 at the protein level (Fig. 4*F*). These results suggest that the increase in observed Nrf2 protein is due to post-translational modification, whereas the increase in observed HO-1 protein is due to *de novo* biosynthesis. The phosphorylation of the Ser-473 site in Akt1 protein is reported to be activated by the Rapamycin-insensitive companion of mammalian target of rapamycin-mTOR complex (mTORC2) (33, 34). To test whether XBP1u or HDAC3 induced Akt1 phosphorylation in a similar manner, the Rapamycin-insensitive companion of mammalian target of rapamycin-mTOR complex inhibitor, AZD2014 (35) was added to Ad-*XBP1u* or Ad-*HDAC3*-infected cells. Cellular fractionation was performed to analyze Akt1 phosphorylation and Nrf2 nuclear translocation. Overexpression of XBP1u or HDAC3 increased Akt1 Ser-473 phosphorylation, the nuclear translocation of phosphorylated Akt1 and Nrf2 and up-regulated HO-1 (Fig. 4*G*). However, in the presence of 5 μmol/liter of AZD2014, all of these effects were diminished (Fig. 4*G*). The presence of XBP1u or HDAC3-induced pAkt1 Ser-473 in the nucleus was confirmed by immunofluorescence staining. This was significantly attenuated by AZD2014 (Fig. 4*H*). The presence of AZD2014 also abolished flow-induced Nrf2 nuclear translocation (Fig. 4*I*). These results suggest that XBP1 is essential for basal and disturbed flow-induced HO-1 expression via regulation of the Akt1/Nrf2 pathway in a Rapamycin-insensitive companion of mammalian target of rapamycin-mTOR dependent manner.

##### XBP1u Physically Interacts with HDAC3

**FIGURE 5.**
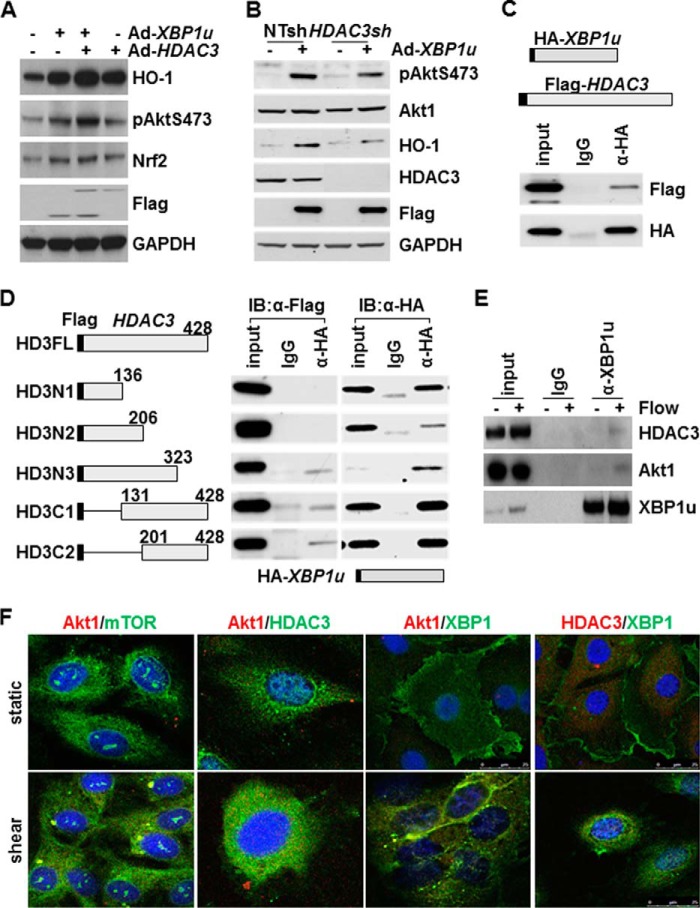
**XBP1 physically interacted with HDAC3.** *A*, XBP1u and HDAC3 synergistically activated HO-1 expression. HUVECs were co-infected with Ad-*XBP1u* and Ad-*HDAC3* at 10 MOI each for 24 h, followed by Western blot analysis. Ad-null virus was included as control and to compensate the MOI. FLAG antibody was used to detect exogenous XBP1u and HDAC3. *B*, knockdown of *HDAC3* via shRNA lentivirus (*HDAC3sh*) attenuated Ad-*XBP1u*-induced HO-1 expression. Non-target shRNA lentivirus (*NTsh*) was included as control. *C*, XBP1u physically interacted with HDAC3. HEK293 cells were co-transfected with HA-*XBP1u* and FLAG-*HDAC3* plasmids, followed by immunoprecipitation with anti-HA antibody and Western blot analysis with anti-FLAG and anti-HA antibodies. *D*, XBP1u bound to amino acid 201–323 region in HDAC3 molecule. The *left panel* indicates the schematic illustration of *HDAC3* truncated mutants. The *right panel* shows the interaction of XBP1u and truncated HDAC3 as revealed by immunoprecipitation assays. *E*, disturbed flow increased XBP1u association with HDAC3/Akt1. Co-immunoprecipitation with anti-XBP1u antibody was performed on static and disturbed flow (4 h)-treated cells, followed by Western blot with anti-HDAC3 or Akt1 and anti-XBP1u antibodies. *F*, disturbed flow induced mTOR/Akt1/HDAC3/XBP1u complex formation in the cytoplasm. Double immunofluorescence staining was performed on static and disturbed flow (4 h)-treated cells. Antibodies are indicated with *red* or *green letters* reflecting the color in the images. Data presented are representatives of three independent experiments.

As described above, both XBP1u and HDAC3 up-regulate HO-1 expression, whereas flow-induced HDAC3 is XBP1-dependent. Therefore, we hypothesized that there was cross-talk between XBP1u and HDAC3 during the regulation of HO-1. To test this, co-expression of HDAC3 and XBP1u was first introduced into HUVECs by co-infection with two viruses. As shown in Fig. 5*A*, overexpression of either XBP1u or HDAC3 alone up-regulated Akt1 phosphorylation, Nrf2 and HO-1, whereas co-expression of XBP1u and HDAC3 had a synergistic effect. Further experiments revealed that knockdown of *HDAC3* attenuated XBP1u-induced Akt1 phosphorylation and HO-1 expression (Fig. 5*B*). Co-immunoprecipitation assays revealed that XBP1u physically bound to HDAC3 in transfected cells (Fig. 5*C*). Using truncated HDAC3 mutants, the binding domain in HDAC3 molecule could be defined to the amino acid 201∼323 region (Fig. 5*D*). Immunoprecipitation with antibody against endogenous XBP1u revealed that XBP1u bound to HDAC3 and Akt1 under disturbed flow (Fig. 5*E*). Double immunofluorescence staining showed that mTOR/Akt1, Akt1/HDAC3, Akt1/XBP1u, and HDAC3/XBP1u co-localized in the cytoplasm (Fig. 5*F*). These results suggest XBP1u/HDAC3/Akt1/mTOR may form a complex to regulate Akt1 phosphorylation, leading to Nrf2 stabilization and HO-1 expression.

## DISCUSSION

The maintenance of redox homeostasis is essential for cell survival and normal cellular functions. It is well known that disturbed flow can activate oxidative stress, which is proatherogenic. In this study, we demonstrate that disturbed flow can also activate anti-oxidative effects via the up-regulation of HO-1 protein in an XBP1u/HDAC3/Akt1/Nrf2 pathway-dependent manner. XBP1u, HDAC3, Akt1, and mTOR may form a complex, which provides a novel mechanism in regulating HO-1 expression, leading to cell survival under oxidative stress.

*HMOX-1* belongs to a group of antioxidant response element (ARE)-regulated genes. The ARE in the promoter of *HMOX-1* is also a shear stress response element, through which the transcription is up-regulated by hemodynamic forces, including laminar shear stress (32, 36–38), oscillatory shear stress (32, 37), and cyclic stretch (39) in ECs and/or smooth muscle cells. Laminar shear stress is more effective as compared with oscillatory shear stress, but the latter seems more effective over long time periods (32, 37). The ARE-regulated *HMOX-1* expression is mediated by the antioxidant transcription factor Nrf2 (40). Shear stress activates Nrf2 posttranslational stabilization and nuclear localization (9, 41). Several signal pathways have been reported to stabilize Nrf2 (42), one of which is PI3K/Akt (31). It is well known that shear stress activates Akt phosphorylation. Thus, flow-induced Akt phosphorylation may be responsible for Nrf2 stabilization, leading to HO-1 up-regulation. However, the signal between the shear stress sensor and Akt/Nrf2 remains unclear. In this study, we observed that oscillatory flow up-regulated XBP1u, HDAC3, Akt phosphorylation, and Nrf2 and HO-1 protein levels, of which the latter four depended on the presence of XBP1. Knockdown of *XBP1* via shRNA lentiviral infection not only abolished flow-induced but also decreased the basal level of Akt1 phosphorylation and HO-1 expression. In contrast, overexpression of XBP1u induced Akt1 phosphorylation and HO-1 up-regulation at mRNA and protein levels. XBP1u does not affect the mRNA level of Nrf2, but increased its protein level potentially through posttranslational stabilization. Indeed, in the presence of transcription and translation inhibitors, disturbed flow still increased Nrf2 protein. Knockdown of Nrf2 abolished flow-induced HO-1 expression. These results suggest that XBP1 may play a fundamental role in HO-1 expression in an Nrf2-dependent way. There are multiple mechanosensors on the cell surface, which transform the mechanical forces into cellular signaling. One of these sensors is the VEGF receptor, which can be activated by flow in a ligand-independent manner (43). In the present study, we found that VEGF receptor inhibitor SU5416 abolished flow-induced XBP1u up-regulation. Therefore, it is possible that XBP1u functions as a signal transducer between the mechanosensor, VEGF receptor, and the Akt1/Nrf2/HO-1 pathway.

Oxidized lipids are a known atherosclerosis risk factor, triggering oxidative stress and ER stress. The three ER stress signal pathways IRE1α/XBP1 splicing, ATF6, and PERK phosphorylation are activated with concomitant up-regulation of HO-1 (44, 45), whereas the up-regulation of HO-1 may inhibit ER stress-triggered EC apoptosis (46). Concerning the ER stress response, most studies have focused on the XBP1 splicing event, the role of XBP1u has been underestimated. In the present study, we found that XBP1u is involved in the basal level expression of HO-1 in cultured ECs and responsible for flow-induced HO-1 up-regulation and that overexpression of XBP1u could induce HO-1 expression. Under ER stress, the activation of ATF6 can trigger XBP1 transcription, leading to the increase of both XBP1u and XBP1s (10, 11, 47). Therefore, a protective role of XBP1u via HO-1 in ER stress may be studied.

Our previous study demonstrated that HDAC3 protects ECs from oxidative stress via Akt phosphorylation (19). In this study, we found that overexpression of HDAC3 could stabilize Nrf2 and up-regulate *HMOX-1* transcription. HO-1 may be the final effector for antioxidant protection. Flow-induced HDAC3 and Akt1 phosphorylation is XBP1-dependent, whereas XBP1u-induced Akt1 phosphorylation and HO-1 expression could be attenuated by HDAC3 knockdown. Moreover, XBP1u and HDAC3 had synergistic effect on Akt1 phosphorylation and HO-1 expression. However, the flow-up-regulated XBP1u and HDAC3 can be attenuated by the presence of PI3K/Akt inhibitor Ly294002. All of these data imply that XBP1u, HDAC3, and Akt1 may form a complex to regulate Nrf2 stabilization and HO-1 expression. Indeed, Akt1 binds to the amino acid 131–201 region in HDAC3 molecule (19), whereas XBP1u binds to the amino acid 201–323 region. Immunoprecipitation with anti-XBP1u antibody could pull down both HDAC3 and Akt1, whereas double immunofluorescence staining revealed that these three molecules co-localized in the cytoplasm. Importantly, the complex may include mTOR. It has been reported that the phosphorylation of Akt serine 473 site is activated by mTORC2 (33, 34). In our study, the mTORC2 inhibitor, AZD2014, abolished XBP1u- or HDAC3-induced Akt Ser-473 phosphorylation, Nrf2 nuclear translocation, and HO-1 expression, suggesting that XBP1u or HDAC3-induced Akt Ser-473 phosphorylation is mediated by mTORC2. Furthermore, disturbed flow induces mTOR and Akt1 co-localization in the cytoplasm. HDAC3 may function as a bridge to bring together XBP1u, Akt1, and mTORC2 to form a complex, leading to XBP1u/HDAC3 stabilization and Akt1 phosphorylation. Our studies indicate that HDAC3 plays a positive role in the regulation of HO-1 expression and in protecting ECs under oxidative stress. A recent report from Su *et al.* (48) showed that sulforaphane-induced HO-1 expression with a decrease in HDAC activity and the protein levels of HDAC1–4 in skin cells, suggesting a negative role of HDAC3 in HO-1 expression. The discrepancy may be due to the different cell models and the stimuli used.

**FIGURE 6.**
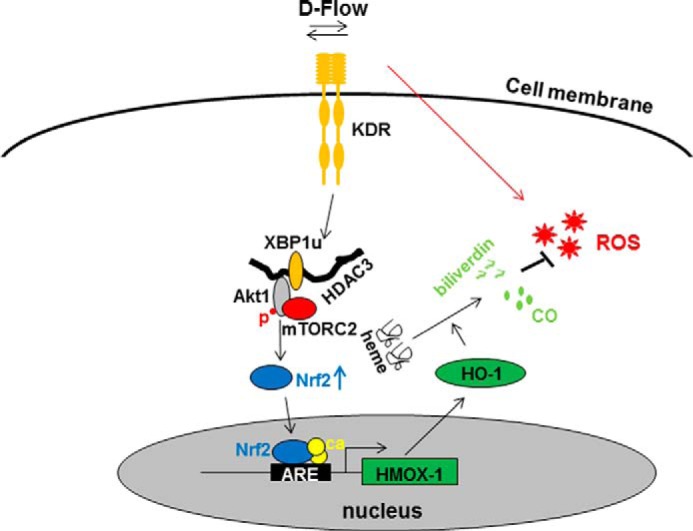
**A schematic illustration of flow-induced HO-1 expression.** Disturbed flow (*D-Flow*) may activate VEGF receptor (*KDR*) in a ligand-independent manner, which in turn induces the complex formation among mTOR, Akt1, XBP1u, and HDAC3. The complex formation stabilizes both XBP1u and HDAC3 and activates Akt1 phosphorylation (*p*), leading to Nrf2 stabilization. Nrf2 translocates into nucleus and binds to the ARE in the *HMOX-1* gene promoter and recruit co-activators (*ca*), promoting the *HMOX-1* transcription. HO-1 catalyzes the heme degradation, which produces antioxidant biliverdin and carbon monoxide (*CO*), antagonizing disturbed flow-induced reactive oxygen species (*ROS*), leading to the maintenance of the redox homeostasis.

In summary, disturbed flow may activate the VEGF receptor in a ligand-independent manner, which in turn induces the formation of a complex among mTORC2, Akt1, XBP1u, and HDAC3. The formation of this complex stabilizes both XBP1u and HDAC3 and activates Akt1 phosphorylation, leading to Nrf2 stabilization. Nrf2 translocates into the nucleus and binds to the ARE in the *HMOX-1* gene promoter, promoting *HMOX-1* transcription. HO-1 catalyzes heme degradation and produces the antioxidant biliverdin and carbon monoxide. Through these mechanisms, ECs protect themselves from disturbed flow-induced oxidative stress, therefore maintaining the redox homeostasis (Fig. 6).

## Footnotes

4 The abbreviations used are:

EC: endothelial cell
HO-1: heme oxygenase 1
XBP1: X-box-binding protein 1
HDAC3: histone deacetylase 3
XBP1u: unspliced XBP1
mTORC2: mammalian target of rapamycin complex 2
IRE1α: inositol-requiring enzyme 1 α
ER: endoplasmic reticulum
XBP1s: spliced XBP1
DMSO: dimethyl sulfoxide
HUVEC: human umbilical vein EC
MOI: multiplicity of infection
Luc: luciferase
ARE: antioxidant response element
Ad: Adenovirus.
